# Analysis of Volatile Compounds in Sea Bass (*Lateolabrax japonicus*) Resulting from Different Slaughter Methods Using Electronic-Nose (E-Nose) and Gas Chromatography-Ion Mobility Spectrometry

**DOI:** 10.3390/molecules26195889

**Published:** 2021-09-28

**Authors:** Yueqi Wang, Jinxing Li, Yanyan Wu, Shengyuan Yang, Di Wang, Qiang Liu

**Affiliations:** 1 Key Lab of Aquatic Product Processing, Ministry of Agriculture and Rural Affairs of the People’s Republic of China, National Research and Development Center for Aquatic Product Processing, South China Sea Fisheries Research Institute, Chinese Academy of Fishery Sciences, Guangzhou 510300, China; wangyueqi@scsfri.ac.cn (Y.W.); lijxgd@163.com (J.L.); wangdi1991624@hotmail.com (D.W.); 2 Collaborative Innovation Center of Seafood Deep Processing, Dalian Polytechnic University, Dalian 116034, China; 3 College of Food Science and Technology, Guangdong Ocean University, Zhanjiang 524088, China; 4 College of Food Science and Engineering, Lingnan Normal University, Zhanjiang 524048, China; 5 Zhuhai Qiangjing Food Co., Ltd., Zhuhai 519100, China; liuqiang052266@126.com

**Keywords:** gas chromatography-ion mobility spectrometry (GC-IMS), electronic nose (E-nose), sea bass, slaughter method, fingerprint, principal component analysis (PCA)

## Abstract

Sea bass (*Lateolabrax japonicus*) is known for its unique flavor and high nutritional value. In this study, the influence of slaughter methods on the volatile compounds (VOCs) in sea bass was investigated using electronic nose (E-nose) technology and gas chromatography-ion mobility spectrometry (GC-IMS). VOCs in raw and cooked sea bass resulting from different slaughter methods were effectively distinguished using both techniques. Aldehydes, ketones, and alcohols were associated with the basic flavor of sea bass, whereas esters, organic acids, and furans enriched the aroma. In raw sea bass, the fishy odor was the strongest in the HSD group (head shot control death), followed by that in the IFD (ice faint to death) and BDS (bloodletting to death) groups. The VOC content increased and stabilized after steaming, enhancing pleasant odors such as fatty and fruity aromas. In cooked sea bass, the content of diacetyl and ethanol was the highest in the EAD group (eugenol anesthesia to death), which may be a residue of eugenol, imparting a distinct irritating chemical odor. Furthermore, abundant (*E*)-2-octenal, 2-heptanone, benzaldehyde, and esters in the BDS group imparted a strong, pleasant aroma. The findings indicate that heart puncture and bloodletting is the preferred slaughter method to maintain sea bass quality, providing new insights into the volatile changes in sea bass induced by different slaughter methods.

## 1. Introduction

Sea bass (*Lateolabrax japonicus)* is a marine species of high commercial value. It inhabits tropical and subtropical regions and is mainly distributed in the Mediterranean Sea, Atlantic Ocean, and near-shore waters of the Bohai Sea, Yellow Sea, and other areas around China [1]. With continuous improvements in living standards, consumers’ demand for healthy diet is increasing. Sea bass has become popular owing to its tender muscle, unique flavor, and high nutrient content, including proteins, vitamins, minerals, and omega-3 fatty acids, such as eicosapentaenoic acid (EPA) and docosahexaenoic acid (DHA).

Flavor is one of the main factors that attracts consumers to certain foods [2]. Volatile compounds (VOCs) in fish are generated from enzymatic reactions, lipid oxidation, and microbial metabolism, and they interact to form the overall flavor [3]. The latter is affected by a complex network of factors, including fish development stage, breeding environment, pre-slaughter fasting, slaughter method, and transportation conditions. The slaughter method is an essential and indispensable factor in meat processing. Improper slaughter induces a strong stress response in the fish, which causes changes in metabolism, blood composition, osmoregulation, and enzyme activity, leading to undesired results (e.g., early rigor mortis, high water loss, severe protein denaturation, and lipid oxidation) that severely affect both quality and flavor [4,5,6]. Secci et al. [7] studied the changes in fatty acid concentrations in farmed rainbow trout due to different slaughter methods. They found a tendency towards decreased free EPA, arachidonic acid (AA), and DHA concentrations in the asphyxiated group, compared with those in the percussion-slaughtered group. Zhang et al. [8] evaluated the effect of the slaughter method on silver carp at 72 h post-mortem and reported a higher level of protein oxidation in the gill cut and stunning groups than in the ice immersion group. However, to date, limited studies have evaluated the effect of the slaughter method on volatile compounds.

Currently, volatile flavors are explored using electronic nose (E-nose) technology, gas chromatography-mass spectrometry (GC-MS), and gas chromatography-ion mobility spectrometry (GC-IMS). A gas-sensitive sensor array derived from E-nose was developed to mimic a mammalian nose, but it can only discriminate overall flavors and cannot specifically describe the dynamics of overall VOCS [9,10]. Currently, GC-MS is the most commonly used instrument for analyzing volatile compounds in the food science field. GC-IMS, a detection technique for analyzing volatile compounds in samples, has emerged in recent years. It is a complementary method used to analyze food VOCs by combining the separation of gas chromatography with the quantitative and rapid advantages of ion mobility spectrometry, which has the advantages of rapid detection and easy operation [11]. GC-IMS combined with E-nose technology enables intuitive comparisons among samples based on establishing a fingerprint and radar chart of VOCs. In the present study, we aimed to use a combination of E-nose and GC-IMS technology to establish volatile profiles and monitor VOC dynamic in sea bass caused by different slaughter methods. This study is expected to provide strategies for monitoring the quality of sea bass.

## 2. Results

### 2.1. E-Nose Analysis

**Figure 1:**
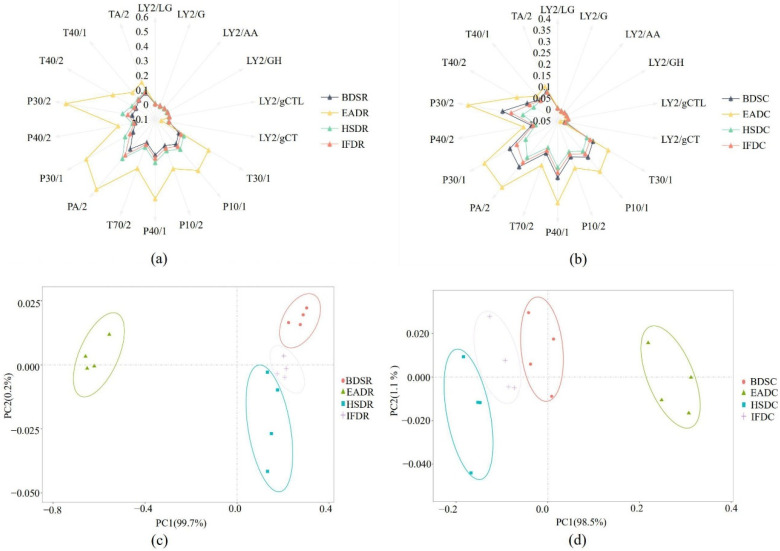
Radar plots (**a**,**b**) and principal component analysis (PCA) plots (**c**,**d**) of raw (R) and cooked (C) sea bass subjected to different slaughter methods. IFD, ice faint to death; HSD, head shot control stun death; EAD, eugenol anesthesia to death; BDS, bloodletting to death.

The P30/2, T40/2, P30/1, PA/2, T70/2, P40/1, P10/2, P10/1, and T30/1 sensors displayed stronger responses to VOCs in sea bass samples than the other sensors, suggesting that the E-nose effectively distinguished sea bass samples in the different treatment groups (Figure 1a,b). Among them, the distinguishing effect was the strongest for the P30/2, P30/1, PA/2, P10/1, and T30/1 sensors. The P30/2 sensor (sensitive to alcohol, combustion products, aldehydes, and hydrogen sulfide) displayed strong responses, indicating increased alcohol and hydrocarbon content. Taken together, the sensors displayed stronger responses for the raw groups than the cooked groups. The principal component analysis (PCA) plots further confirmed the results displayed in the radar graphs. The PCA plot of VOCs in the raw groups is shown in Figure 1c. PC1 and PC2 comprised 99.7% and 0.2% of the PCA distribution area, respectively, explaining 99.9% of the total variation and indicating that PC1 and PC2 sufficiently represented most of the data. The EADR group was clearly separated from the other groups, whereas the BDSR group was differentiated from the HSDR and IFDR groups. The PCA plot of VOCs in the cooked groups is shown in Figure 1d. PC1 and PC2 comprised 98.5% and 1.1% of the PCA distribution area, respectively, with a cumulative variance contribution rate of 99.6%, indicating that PC1 and PC2 represented most of the original data. The EADC group was clearly separated from the other three cooked groups and was farthest from the HSDC group, indicating that some compounds were altered during cooking.

### 2.2. Sensory Evaluation

The sensory evaluation results are shown in Table 1. There was an obvious difference among the different slaughter samples, suggesting that the slaughter methods had an effect on the flavor. The EAD group had the lowest flavor score and the lowest comprehensive scores due to residual eugenol. The HSD and IFD groups had a strong fishy odor, whereas the BDS group had a strong, pleasant aroma. Therefore, the BDS group had the highest flavor score. Regarding texture, the BDS group was more tender and elastic than the other groups. The BDS group had a weak pre-slaughter stress response, exhibited low muscle glycogen consumption, and presented a high lactic acid content, resulting in a strong enzyme activity required for the meat ripening process. As a result, protein degradation was greater, and the meat was more tender in the BDS group. The IFD group exhibited less tender and less elastic meat as a result of the contraction and shortening of myofibrils at low temperatures. Therefore, the sensory score of the IFD texture was low. The HSD and EAD groups presented increased ATP consumption, leading to necropsy due to acute stress, thereby resulting in poor tenderness and poor elasticity. It can be preliminarily considered that heart puncture and bloodletting is more suitable for sea bass.

### 2.3. Analysis of GC-IMS Compositional Spectra and Profile Differences

**Figure 2:**
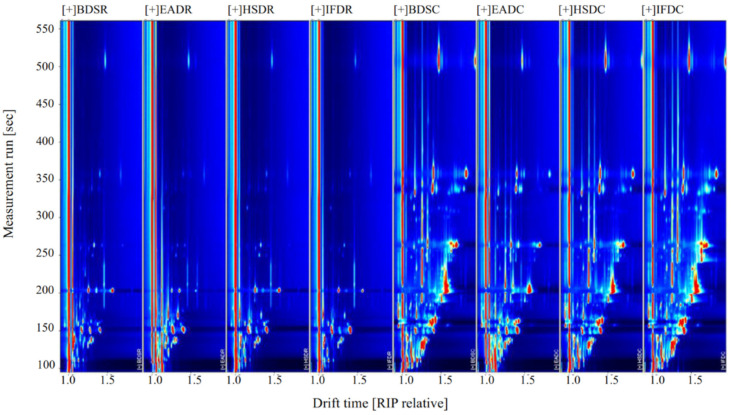
Ion migration spectra from gas chromatography-ion mobility spectrometry (GC-IMS) of raw (R) and cooked (C) sea bass subjected to different slaughter methods. IFD, ice faint to death; HSD, head shot control stun death; EAD, eugenol anesthesia to death; BDS, bloodletting to death.

Figure 2 shows the compositional spectra of VOCs in sea bass samples in the different treatment groups. The ordinate and abscissa represent the retention time and migration of the gas phase, respectively, and each reactive ion peak corresponds with a volatile substance. Generally, the red color and larger spot areas in the spectra indicate higher VOC content, whereas lighter colors and smaller areas indicate lower VOC content. A comparison of the GC-IMS profiles of the eight treatment groups revealed an increase in VOC type and content in cooked samples. The VOC content significantly differed between the raw and cooked groups. Some VOCs with large differences were considered fingerprint characteristic reference points to distinguish the slaughter methods.

**Figure 3:**
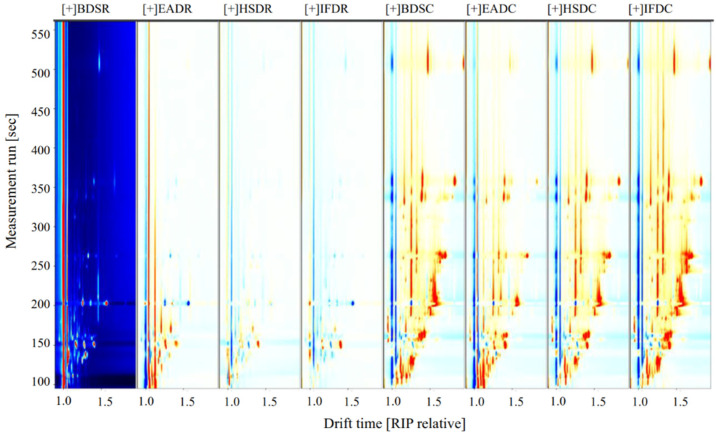
Volatile compound component spectra of raw (R) and cooked (C) sea bass subjected to different slaughter methods, using the BDSR group as the reference. IFD, ice faint to death; HSD, head shot control stun death; EAD, eugenol anesthesia to death slaughter; BDS, bloodletting to death slaughter.

To compare the differences in VOCs among the treatment groups, the BDSR group was used as a reference (Figure 3). The blue areas indicate a lower VOC content in the BDSR group than in the reference sample, whereas the red areas indicate a higher VOC content. Darker colors indicate a greater difference from the reference. More red spots were observed in the retention time range of 100–500 s for the EADR group than for the BDSR group, but there were fewer red and blue spots in the HSDR and IFDR groups, which was consistent with the results of the PCA analysis. The VOC content in the cooked groups was generally higher than that in the reference BDSR group. However, some VOC signals in the EADC group were weak or had disappeared, compared with those in the other cooked groups.

### 2.4. Identification of VOCs

**Figure 4:**
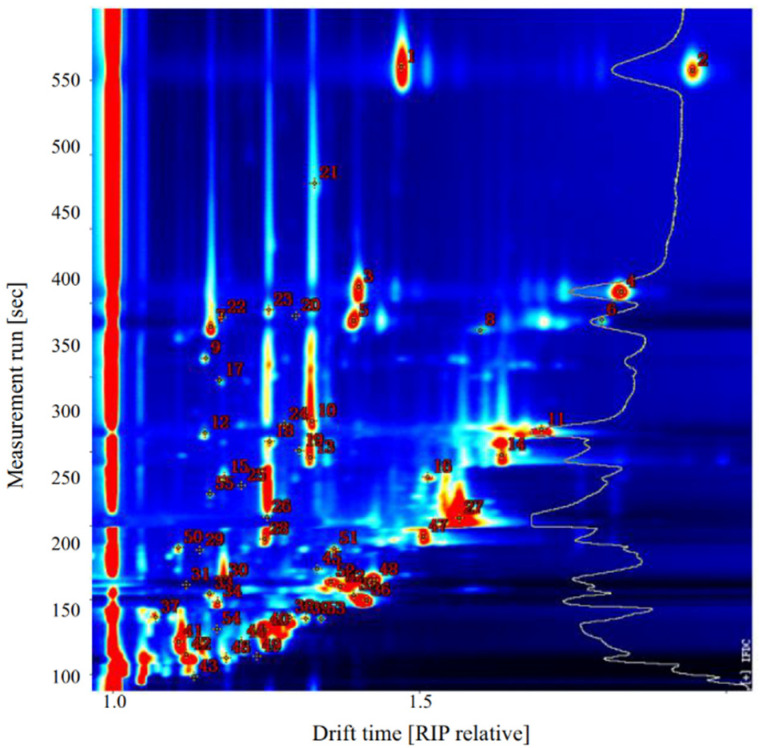
Representative topographic plot of gas chromatography-ion mobility spectrometry (GC-IMS) spectra with selected markers acquired from sea bass.

Qualitative analysis of VOCs in sea bass samples was performed using the NIST and IMS databases provided with GC-IMS software. As shown in Figure 4 and Table 2, 31 VOCs were identified in raw sea bass, including seven aldehydes, four alcohols, one hydrocarbon, six ketones, three esters, two acids, one furan, one sulfur-containing compound, and six unidentified compounds.

**Figure 5:**
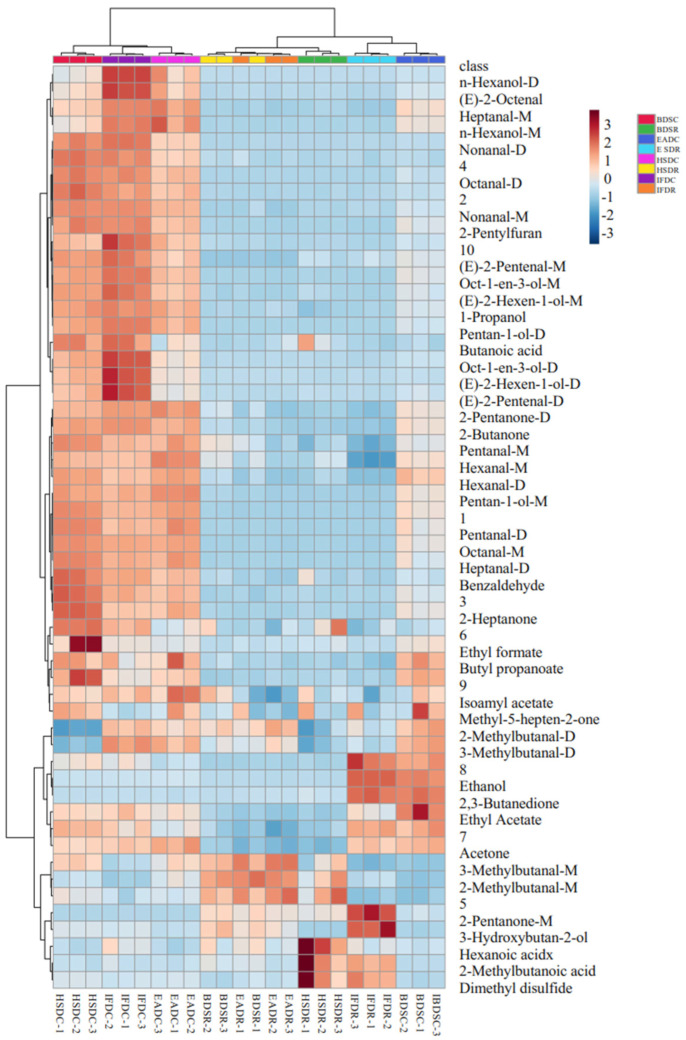
Heat map visualization of flavor-imparting compounds in raw (R) and cooked (C) sea bass subjected to different slaughter methods. IFD, ice faint to death; HSD, head shot control stun death; EAD, eugenol anesthesia to death slaughter; BDS, bloodletting to death slaughter.

Changes in the VOC levels among the treatment groups were visualized in the fingerprints represented as a heatmap (Figure 5), where each row represents a VOC in the sample and each column represents the VOC signal in the same sample. The VOC type and content varied across the raw groups, depending on the slaughter method. The EADR group had the highest VOC content among the raw groups. Five VOCs, namely acetone, ethanol, 3-hydroxybutan-2-one, ethyl acetate, and 2,3-butanedione, were significantly more abundant in the EADR group than in the other raw groups, but the hexanal content was significantly lower in the EADR group. Ethanol and 2,3-butanedione were not detected in the HSDR, IFDR, and BDSR groups. The BDSR group had higher hexanoic acid, 2-pentylfuran, methyl-5-hepten-2-one, and 2-methylbutanoic acid levels than the other raw groups, whereas 3-hydroxybutan-2-one was not detected. The HSDR group had higher benzaldehyde, pentan-1-ol, 2-butanone, 1-octen-3-ol, pentanal, and isoamyl acetate levels than the other raw groups. The 2-methylbutanal, 3-methylbutanal, and 1-propanol were higher in the HSDR and IFDR groups than in the other raw groups. Furthermore, there were significant differences in the monomer and dimer content of the same compound. The 2-pentanone monomer and dimer levels were higher and lower in the EADR group, respectively, than in the HSDR group.

The diversity of VOCs in sea bass samples significantly increased with steaming, as shown in Figure 5. In cooked seabass, 39 VOCs were detected, including 9 aldehydes, six alcohols, one hydrocarbon, six ketones, three esters, two acids, one furan, one sulfur-containing compound, and 10 unidentified compounds. Two aldehydes [(*E*)-2-octenal and (*E*)-2-pentenal] and two alcohols [(*E*)-2-hexen-1-ol and *n*-hexanol] were identified after steaming; they were not detected in raw sea bass. The composition of ketones (2-heptanone appeared, 3-hydroxybutan-2-one degraded), esters (butyl propanoate appeared, isoamyl acetate degraded), and acids (butanoic acid appeared, 2-methylbutanoic acid degraded) changed, but their relative quantities remained unchanged. In contrast to their levels in the raw groups, the hexanal, ethyl formate, ethyl acetate, benzaldehyde, butyl propionate, 2-butanone, acetone, hexanoic acid, benzaldehyde, 1-propanol, 2-pentylfuran, and methyl-5-hepten-2-one levels were elevated in all cooked groups. The dimethyl disulfide level detected in the EADR and BDSR groups decreased after steaming. The 2-pentanone monomer content was higher in the raw groups than in the cooked groups, and its dimer content increased after steaming. Some compounds, namely heptanal, nonanal, pentanal, octane, (*E*)-2-hexen-1-ol, and pentan-1-ol, were present only as monomers in the raw groups but were identified as high-content dimers and monomers after steaming.

### 2.5. Effects of Slaughter Method on the Changes in VOCs in Raw and Cooked Sea Bass

**Figure 6:**
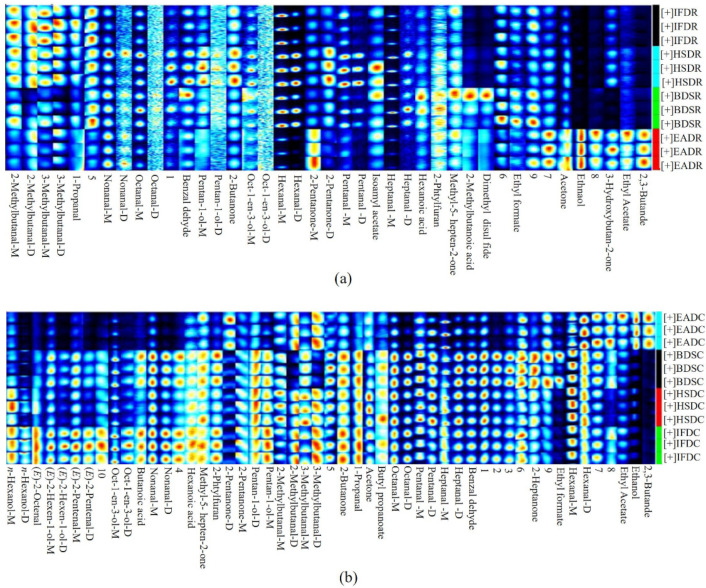
Gallery plot of selected signal peak areas obtained from raw (**a**) and cooked (**b**) sea bass obtained using different slaughter methods. IFD, ice faint to death; HSD, head shot control stun death; EAD, eugenol anesthesia to death; BDS, bloodletting to death; C, cooked; R, raw.

The differences in volatile compounds between the raw and cooked groups are shown in Figure 6. Aldehydes can affect the overall flavor of foods owing to their lower thresholds and relatively higher abundance [12]. Hexanal contributes to fresh grass-like and fishy odors and is considered an oxidation product of linoleic acid and AA [13]. Nonanal and heptanal, as secondary oxidation products of oleic acid, also impart a fishy odor [14]. Here, the levels of hexanal, nonanal, and heptanal were generally high in the raw groups, with the highest content in the HSDR group, followed by the IFDR and BDSR groups. The HSDR group had the strongest fishy odor, likely because HSD generated a larger stress response in the fish, resulting in increased lipid oxidase activity, enhanced lipolytic activity, increased formation of lipid peroxidation metabolites, and eventually elevated levels of aldehydes. This result corroborated that of Resconi et al. in beef [15].

Thermal processing promotes the release of VOCs [16]. A comparison of VOCs in the raw and cooked groups, obtained using the same slaughter method, IFD, induced the highest increase in the nonanal and heptanal levels, whereas HSD induced the greatest increase in the hexanal levels. However, the mechanism underlying this phenomenon remains unclear. These findings suggest that a stronger fishy odor was present in the IFDC and HSDC groups than in their raw counterparts. The quantities of VOCs, such as nonanal, hexanal, and heptanal, in the EADC group were significantly lower than those in the other cooked groups, which was consistent with the findings of a previous study, which reported that eugenol inhibited the production of VOCs during the refrigeration of grass carp [17]. 2-Methylbutanal and 3-methylbutanal are Strecker aldehydes, derived from the catabolism of branched-chain amino acids, which contribute to malt, caramel, and chocolate flavors [18,19]. Except in the EAD group, the 2-methylbutyraldehyde content was higher in the raw groups than in the cooked groups using the same slaughter method, indicating that the compound was degraded upon steaming. In addition to saturated aldehydes, unsaturated aldehydes were also detected. Benzaldehyde is generated from the oxidation of linoleic acid and imparts nutty, bitter almond, and cherry flavors. Benzaldehyde and other volatile compounds are present in 75% of VOCs in grass carp [20]. The benzaldehyde content increased in all cooked groups, as steaming promoted lipid oxidation and phenylethyl aldehyde oxidation [21,22]. Notably, the differences in volatile compounds among the cooked groups were related to the differences in lipid-derived compounds in the raw groups. The benzaldehyde content was the highest in the BDS group, in both raw and cooked samples. (*E*)-2-Pentenal and (*E*)-2-octenal were produced after steaming. (*E*)-2-octenal imparts a broth flavor and its content was in the following order: IFDC > HSDC > BDSC > EADC. The aldehyde content was the highest in the IFD group, followed by the HDS group. A large amount of blood was obtained in the IFD and HSD groups without bloodletting treatment. A good correlation has been found between hemoglobin content and lipid oxidation. Hemoglobin dilution occurs when hemoglobin leaks out of the red blood cells. Hemoglobin dilution promotes the formation of hemoglobin subunits, which accelerates hemoglobin autoxidation and heme release. Thus, hydroxyl radicals generated from the reaction of pro-oxidants (hemoglobin and heme) with hydrogen peroxide accelerated lipid oxidation [23].

Ketones are products of fatty acid oxidation. Ketones with low thresholds enhance or modify fishy odors by interacting with aldehydes or other compounds [24]. The ketone content in the raw groups was in the following order: EAD > HSD > IFD > BDS, indicating that BDS better maintained mild and pleasant flavors than the other slaughter methods. 2,3-Butanedione accounted for 19.8% of ketones in the EADR group, whereas it was not detected in the other raw groups. Therefore, 2,3-butanedione was presumed to have originated from the anesthetic. The enzymes in fish blood promoted the reaction between lipid and/or protein in the muscle of the HSDR and IFDR groups, which in turn increased the ketone content and enhanced the fishy odor. Steaming further affected the differences in lipid-derived metabolites. The levels of concentration of 2-pentanone, propanol, and 2-butanone was higher in the cooked groups than in the raw groups; however, the levels of 2-pentanone showed a reverse trend in the EAD group. 2-Heptanone imparts a gravy-like aroma and is generated by lipid oxidation [25]. The 2-heptanone content was generally the highest in the BDSC group, followed by the HSDC and IFDC groups. The VOC levels in the raw groups could also explain the differences observed in the cooked groups. The 2,3-butanedione content was reduced slightly after steaming; however, it was present in excessively high amounts in the EADR and EADC groups, resulting in strong creamy, buttery, and caramel flavors [26].

Alcohols are derived from the degradation of secondary hydroperoxides of fatty acids, or the reduction in carbonyl compounds [27]. 1-Octen-3-ol, the dominant alcohol imparting mushroom-like and earthy odors, enhanced the fishy taste of aldehydes. Generally, 1-octen-3-ol is generated by the degradation of linoleic acid and AA. A higher 1-octen-3-ol content was observed in the HSDR and BDSR groups, and the lowest content was observed in the EADR group. Grigorakis et al. reported that the AA level in wild gilthead sea bream was significantly higher than that in cultured gilthead sea bream, showing a positive correlation with 1-octen-3-ol content [28,29]. This observation suggests that the degradation of AA was higher in the HSDR and BDSR groups than in the IFDR and EADR groups. Steaming amplified the differences in VOCs induced by different slaughter methods. The lowest 1-octen-3-ol content was present in the EADC group, equivalent to one-third of that in the IFDC group; thus, the earthy odor was stronger in the IFDC group. Saturated alcohols, such as hexanol, 1-pentanol, and 1-propanol are mostly detected in steamed crustaceans and fish [30]. Hexanol was the most abundant in the IFDC group after steaming and contributed to sweet and fruity aromas. 1-Pentanol, a product of linoleic acid oxidation, contributed an herbaceous aroma, which was further intensified after steaming [31,32]. (*E*)-2-Hexenol, another product generated after steaming, imparted a fruity aroma. Both 1-pentanol and (*E*)-2-hexenol levels were the highest in the IFDC group, followed by those in the BDSC group. However, the threshold value of unsaturated alcohols was lower than that of saturated alcohols; hence, (*E*)-2-hexenol contributed more to the flavor of sea bass [33].

The anesthetic used in the study contained a large amount of ethanol. High concentrations of 2,3-butanedione and ethanol contributed to strong and unpleasant odors in the EAD group (strong floral, vanilla, and woody flavors). In addition to that observed in the EADR group, the alcohol content increased in the three raw groups as the stress level of the fish increased. Stronger stress levels at slaughter lead to more extensive glycogen degradation and higher alcohol content in sea bass [34]. The alcohol content in the raw groups decreased as follows: HSD > IFD > BDS. As the BDSR group was not subjected to severe stimulation during slaughter, this group retained a pleasant aroma. The highest alcohol content was observed in the HSDR and IFDC groups, which may be attributed to the absence of bloodletting with the IFD and HSD methods. Before cooking, the low lipid oxygenase activity in the IFD group generated less alcohol, owing to low temperature slaughter, whereas more alcohol was generated by thermal oxidative decomposition of lipids upon steaming.

Esters containing short-chain fatty acids impart a sweet and fruity aroma, whereas those containing long-chain fatty acids impart fatty odors [35]. Esters containing short-chain fatty acids, such as isoamyl acetate, butyl propionate, ethyl formate, and ethyl acetate, were detected in sea bass. The ester content was low in the raw groups, in the following order: EAD > BDS > HSD > IFD. The increase in ester content upon steaming was different from that described by Lorenzo et al. in pony meat, which indicated that significant differences exist in the VOC composition in different kinds of meat [36]. Esters are derived from the esterification of alcohols and carboxylic acid compounds, and the ester content in the cooked groups was in the following order: BDS > EAD > IFD > HSD. The highest ester content was observed in the EADR and BDSC groups. However, the mechanism of this change requires further investigation. Low levels of other VOCs, including dimethyl disulfide, 2-pentylfuran, and octane, differed among the four raw groups and had little effect. As 2-pentylfuran originates from caramelization reactions and thermal degradation of carbohydrates, its content increased significantly after steaming. The 2-pentylfuran content was the highest in the IFDC group, followed by that in the BDSC group. 2-Pentylfuran imparts a green bean-like aroma and contributes to the overall flavor of steamed mussels [37,38]. Octane, derived from the homolysis of fatty acid alkyl radicals, imparts a sweet flavor. The octane content increased several times after steaming. Its highest content was detected in the BDSC group, which enhanced the overall flavor despite the higher threshold value. The results demonstrate that the slaughter method significantly influences both VOC content and type in sea bass, likely due to its effects on the levels of flavor precursors in muscle, such as fatty acids, free amino acids, and sugars. Therefore, the effect of slaughter methods on flavor precursors remains to be evaluated in future studies.

## 3. Materials and Methods

### 3.1. Animals and Ethics Approval

All methods used in this study complied with the Chinese National Guidelines for the use and care of laboratory animals. The animal experiment protocol was approved by the Academic Council of the South China Sea Fisheries Research Institute, Chinese Academy of Fishery Sciences.

### 3.2. Materials

Live sea bass (body length: 25 ± 2.0 cm; weight: 500 ± 10.0 g) were purchased from Vanguard Supermarket (Guangzhou, Guangdong, China). They were transported in large tanks (1.2 m × 1.2 m × 0.8 m). During transport, the stagnant water was saturated using an oxygen diffuser connected by a rubber tube to a liquid oxygen tank and transported to the laboratory within 1 h. Eugenol solution was prepared as follows: eugenol and absolute ethanol were mixed (1:9 *v/v*), and the emulsion was subsequently diluted to 40 mg/L with water. Twelve sea bass were slaughtered using each method: ice faint to death (IFD), head shot control stun death (HSD), eugenol anesthesia to death (EAD), and bloodletting to death (BDS). For IFD, each fish was immersed in a box containing ice and kept in it until the observation of the apparent stunning, at approximately 20 min. For HSD, the fish were hit on the head several times with the back of a knife. Until the occurrence of apparent stunning, the back flesh was collected. For EAD, the fish were immersed in the eugenol solution until anesthesia, which was characterized by the absence of swimming and consciousness. For BDS, the heart of the fish was pierced, leaving a hole for blood drainage.

After slaughtering 12 sea bass in each group in the same way, the back meat of each sea bass was collected and cut into small fish fillets. The back meat of six sea bass was steamed in boiling water for 30 s and used as cooked sample group (C). The cooked samples were minced using a grinding machine (IKA-T25; Germany IKA Co., Ltd., Guangzhou, China), and 30 g of each sample was used for the electronic nose and GC-IMS assay. The remaining samples were used as the raw meat (raw meat group (R)) and was also grinded, weighed, and tested. There were eight groups: HSDR, HSDC, IFDR, IFDC, BDSR, BDSC, EADR, and EADC. All the experiments were performed in triplicate, except for the electronic nose test, which was repeated four times.

### 3.3. E-Nose Analysis

The dynamic headspace method for VOC analysis was adapted from Wang et al. [39], with slight modifications. We determined the overall odor profiles and performed a qualitative analysis using a Fox 4000 electronic nose (Alpha MOS, Toulouse, France) with 18 sensors (Table 3) [40]. Each sample (1 g) was placed in a bottle with 15 mL headspace and heated at 75 °C for 600 s. The temperature of the injector was 60 °C, and the injection time was 1 s. The injection volume was 250 μL, and clean dry air was used as the injection carrier gas at a flow rate of 150 mL/min. The data acquisition time was 120 s. To remove any remaining sample odor, the device was cleaned with clean dry air for 10 min after each sample. All analyses were performed using four biological replicates.

### 3.4. Sensory Evaluation

The sensory evaluation panelists consisted of a group of experts who have been engaged in the research of sea bass production and processing for a long time, including four males and four females. All samples were numbered with a random three-digit method and presented in random order. The odor and texture of sea bass were evaluated by nine-point scale scoring as shown in Table 4. During the assessment, water was provided to assessors to avoid fatigue and residual effects. All samples were subjected to sensory evaluation in the same laboratory.

### 3.5. GC-IMS Analysis

Volatile compounds present in the samples were identified and quantified using a GC-IMS flavor analyzer (FlavourSpec^®^, Dortmund, Germany), equipped with a syringe and autosampler unit for headspace analysis. Samples (4 g) were placed in a bottle with 20 mL headspace and incubated at 60 °C for 15 min. The injection volume was 500 μL, which was automatically injected into the injector using a heated syringe (85 °C). The sample was then transferred onto an MXT-5 capillary column (15 m × 0.53 mm × 1 μm; Agilent Technologies, Santa Clara, CA, USA) using nitrogen (99.99%) and subjected to the following programmed flow: 2 mL/min, then 2 mL/min for 2 min, followed by 100 mL/min for 18 min. The column was maintained at 60 °C with a drift tube temperature of 45 °C. The drift gas flow was set to a constant flow rate of 150 mL/min. All analyses were performed in triplicate. The final results represent the average of three replicates.

### 3.6. Data Processing

All the experiments were performed in triplicate, except for the electronic nose test, which was repeated four times. All statistical analyses were performed using SPSS version 19.0 (IBM, Armonk, NY, USA). Between-group differences were formally compared using the one-way analysis of variance. All values are presented as mean ± standard deviation/standard error of the mean, and differences were considered statistically significant at *p* < 0.05. Data acquired from E-nose were visualized and analyzed using Alphasoft V14 software (Alphamos, Tolouse, France). The volatile “fingerprint” map of sea bass samples in the different groups was analyzed using the Laboratory Analysis Viewer, two plug-ins and GC-IMS Library Search software, provided by GC-IMS instruments (GAS, Dortmund, Germany). Reporter plug-in compared spectral differences between samples directly (2D top view and difference spectra); Gallery Plot plug-in visually and quantitatively compared the differences in VOCs between samples.

The volatile compounds “fingerprint” map of sea bass samples in the different groups was analyzed using the Laboratory Analysis Viewer and the plug-in GC-IMS Library Search software provided by GC-IMS instruments (GAS, Dortmund, Germany).

## 4. Conclusions

The VOCs of raw and cooked sea bass obtained using different slaughter methods were effectively distinguished and identified using the E-nose technology and GC-IMS. The results demonstrated significantly different VOC profiles for samples prepared with eight different treatments. Aldehydes, ketones, and alcohols were associated with the basic flavor of sea bass, whereas esters, organic acids, and furans enriched the volatile compounds. The slaughter method exerted a strong influence on the volatile profile, and the VOC composition differed before and after cooking. Overall, raw fish had a soft, pleasant, and fruity aroma, accompanied by a fishy odor. Particularly, the fishy odor in the HSDR group was the strongest, followed by that in the IFDR and BDSR groups. The VOC concentration increased and stabilized after steaming, enhancing pleasant odors. The concentration of diacetyl and ethanol was the highest in the EAD group (eugenol anesthesia to death), which may be a residue of eugenol, thus causing a distinct irritating chemical odor, whereas (*E*)-2-octenal, 2-heptanone, benzaldehyde, and esters were abundant in the BDSC group, imparting a strong, pleasant aroma. Our findings suggest that heart puncture and bloodletting is the most suitable slaughter method for sea bass to maintain high quality. This study provides new insights into the volatile changes in sea bass induced by different slaughter methods and provides a theoretical foundation for exploring the optimal slaughter method.

## Figures and Tables

**Table 1:** Sensory rating scores for steaming sea bass obtained using different slaughter methods.

Evaluation Dimension	Steaming Samples			
	EADC	HSDC	IFDC	BDSC
Flavor	3.75 ± 0.90 ^d^	6.06 ± 0.58 ^c^	7.44 ± 0.58 ^b^	8.40 ± 0.48 ^a^
Texture	7.25 ± 0.89 ^b^	7.69 ± 0.84 ^ab^	5.88 ± 0.69 ^a^	8.38 ± 0.58 ^a^
Comprehensive score	5.56 ± 0.78 ^d^	6.81 ± 0.46 ^c^	7.50 ± 0.46 ^b^	8.44 ± 0.56 ^a^

Notes: means in a row without a common superscript letter were different at least at *p* < 0.05.

**Table 2:** Gas chromatography-ion mobility spectrometry (GC-IMS) integration parameters of volatile compounds in sea bass samples.

Count.	Compound	CAS#	RI	Rt (s)	Dt (ms)	Peak Volume							
						HSDR	IFDR	BDSR	EADR	EADC	HSDC	BDSC	IFDC
Aldehydes													
1	Nonanal-M	124,196	1109.7	508.8	1.47	784.3 ± 203.0 ^a^	528.7 ± 86.8 ^b^	815.9 ± 41.9 ^a^	874.8 ± 59.0 ^a^	1400.4 ± 136.2 ^C^	3204.3 ± 12.3 ^B^	3931.2 ± 92.1 ^A^	3924.7 ± 37.7 ^A^
2	Nonanal-D	124,196	1108	506.3	1.95	84.9 ± 22.7 ^a^	72.6 ± 10.5 ^a^	86.6 ± 8.6 ^a^	97.0 ± 9.1 ^a^	172.9 ± 27.6 ^D^	944.5 ± 26.1 ^C^	1548.1 ± 110.1 ^B^	1731.3 ± 39.5 ^A^
9	Benzaldehyde	100,527	957.6	312.7	1.15	116.3 ± 8.6 ^a^	88.5 ± 5.1 ^a^	131.3 ± 51.9 ^a^	99.4 ± 10.9 ^a^	135.7 ± 10.9 ^D^	244.6 ± 12.7 ^C^	325.0 ± 20.5 ^A^	276.1 ± 5.0 ^B^
10	Heptanal-M	111,717	907.7	270.2	1.33	419 ± 23.4 ^a^	297.6 ± 31 ^b^	433.8 ± 56.9 ^a^	232.6 ± 9.5 ^b^	1567.3 ± 148.8 ^B^	2664.4 ± 145.8 ^A^	1872.3 ± 118.2 ^B^	2870.9 ± 28.3 ^A^
11	Heptanal-D	111,717	901.2	264.6	1.70	56.2 ± 9.8 ^a^	32.3 ± 1.6 ^b^	50.6 ± 9.6 ^a^	33.2 ± 4.3 ^b^	796.0 ± 107.4 ^C^	2108.0 ± 261.3 ^AB^	2454.3 ± 76.2 ^A^	1974.6 ± 74.6 ^B^
21	(*E*)-2-octenal	2,548,870	1055.1	430.2	1.33	40.1 ± 7.8 ^ab^	44.4 ± 7.7 ^a^	44.0 ± 4.7 ^a^	31.3 ± 3.4 ^b^	98.3 ± 5.6 ^C^	288.9 ± 73.6 ^B^	211.7 ± 39.9 ^B^	529.0 ± 20.4 ^A^
26	Hexanal-M	66,251	796.1	205.4	1.25	1267.4 ± 34.3 ^a^	925.3 ± 20.7 ^c^	1095.6 ± 159.1 ^b^	432.3 ± 52.2 ^d^	1498.4 ± 54.9 ^C^	2144.9 ± 35.0 ^A^	1842.1 ± 55.0 ^B^	1761.1 ± 17.4 ^B^
27	Hexanal-D	66,251	795.6	205.1	1.57	1741.0 ± 122.3 ^a^	541.2 ± 57.8 ^c^	1255.3 ± 173.5 ^b^	256.6 ± 26.2 ^c^	4895.1 ± 14.4 ^B^	5950.6 ± 164.4 ^A^	6035.4 ± 130.7 ^A^	4906.4 ± 169.7 ^B^
30	Pentanal-M	110,623	694.2	162.5	1.18	594.1 ± 68.9 ^a^	451.9 ± 103.5 ^ab^	389.0 ± 89.5 ^bc^	273.2 ± 35.9 ^c^	584.5 ± 19.4 ^C^	877.4 ± 65.5 ^B^	955.1 ± 19.2 ^A^	822.4 ± 22.1 ^B^
33	2-methylbutanal-M	96,173	665.6	154.1	1.16	535.7 ± 47.1 ^a^	538.5 ± 10.7 ^a^	421.0 ± 116.1 ^a^	268.4 ± 18.8 ^b^	195.7 ± 6.0 ^C^	323.9 ± 28.7 ^A^	280.1 ± 10 ^B^	216.5 ± 11.5 ^C^
34	3-methylbutanal-M	590,863	644.6	148.4	1.17	602.6 ± 13.9 ^b^	734.6 ± 7.2 ^a^	441.1 ± 149.2 ^c^	239.7 ± 19.2 ^c^	275.2 ± 15.3 ^C^	496.7 ± 53.3 ^A^	550.9 ± 21.7 ^A^	340.4 ± 19.1 ^B^
35	2-methylbutanal-D	96,173	662.6	153.3	1.39	1268.0 ± 101.6 ^ab^	1383.0 ± 232.4 ^a^	558.3 ± 283.5 ^c^	938.2 ± 162.6 ^bc^	1497.3 ± 150.8 ^A^	1269.8 ± 92.3 ^B^	361.8 ± 46.3 ^C^	1430.6 ± 59.1 ^AB^
36	3-methylbutanal-D	590,863	649.6	149.8	1.41	1276.6 ± 160.3 ^ab^	1494.3 ± 274.9 ^a^	545.0 ± 106.0 ^c^	995.1 ± 174.9 ^b^	2230.6 ± 153.2 ^AB^	2126.1 ± 188.2 ^B^	536.1 ± 102.3 ^C^	2442.6 ± 12.0 ^A^
48	Pentanal-D	110,623	695.9	163.2	1.43	102.9 ± 9.7 ^a^	41.9 ± 0.7 ^bc^	56.2 ± 12.1 ^b^	39.2 ± 4.0 ^c^	873.1 ± 286.6 ^C^	1860.4 ± 232.2 ^AB^	2064.4 ± 37.4 ^A^	1502.0 ± 100.1 ^B^
50	(*E*)-2-pentenal-M	1,576,870	749.6	185.0	1.11	26.8 ± 3.0 ^b^	22.7 ± 1.3 ^b^	64.2 ± 15.4 ^a^	67.8 ± 12.5 ^a^	95.8 ± 7.5 ^D^	164.0 ± 15.6 ^C^	212.9 ± 4.3 ^B^	240.6 ± 21.5 ^A^
51	(*E*)-2-pentenal-D	1,576,870	748.3	184.5	1.37	8.1 ± 2.4 ^ab^	5.6 ± 0.2 ^b^	12.1 ± 3.6 ^a^	6.8 ± 2.0 ^b^	23.7 ± 6.6 ^C^	51.9 ± 8.6 ^C^	108.6 ± 13.7 ^B^	202.4 ± 26.7 ^A^
Alcohols													
7	oct-1-en-3-ol-M	3,391,864	982.4	333.9	1.16	123.5 ± 11.7 ^a^	100.3 ± 19.1 ^ab^	124.3 ± 17.5 ^a^	91.6 ± 9.6 ^b^	588.5 ± 69.0 ^D^	1181.0 ± 54.3 ^C^	1431.6 ± 41.0 ^B^	1749.6 ± 51.0 ^A^
8	oct-1-en-3-ol-D	3,391,864	979.3	331.2	1.60	40.3 ± 3.2 ^a^	41.9 ± 1.5 ^a^	41.1 ± 3.1 ^a^	36.6 ± 3.4 ^a^	60.0 ± 1.6 ^D^	119.2 ± 16.2 ^C^	189 ± 12.6 ^B^	283.3 ± 14.6 ^A^
13	*n*-Hexanol-M	111,273	870.9	245.5	1.32	60.6 ± 8.6 ^a^	54.5 ± 2.0 ^a^	62.7 ± 8.0 ^a^	53.8 ± 9.3 ^a^	412.5 ± 27.3 ^B^	957.6 ± 206.7 ^A^	467.2 ± 113.2 ^B^	1006.3 ± 21.8 ^A^
14	*n*-Hexanol-D	111,273	874.4	247.4	1.64	41 ± 5.1 ^a^	35.7 ± 5.8 ^a^	37.7 ± 2.2 ^a^	41.2 ± 1.5 ^a^	134.3 ± 23.2 ^C^	928.0 ± 389.2 ^B^	436.3 ± 124.6 ^C^	1820.7 ± 26.5 ^A^
15	(*E*)-2-hexen-1-ol-M	928,950	846.7	232.6	1.18	60.6 ± 8.2 ^a^	62.4 ± 7.6 ^a^	76.5 ± 11.8 ^a^	61.4 ± 0.4 ^a^	119.3 ± 11.9 ^D^	214.7 ± 9.2 ^C^	266.2 ± 2.9 ^B^	313.7 ± 19.6 ^A^
16	(*E*)-2-hexen-1-ol-D	928,950	846.7	232.6	1.52	25.16 ± 4.9 ^a^	25.56 ± 1.3 ^a^	17.98 ± 0.9 ^b^	21.96 ± 3.8 ^ab^	34.2 ± 4.9 ^D^	134.8 ± 17.6 ^C^	249.4 ± 23.5 ^B^	435.7 ± 52.8 ^A^
28	pentan-1-ol-M	71,410	764	190.9	1.25	99.6 ± 10.9 ^a^	55.6 ± 8.4 ^b^	58.1 ± 10.5 ^b^	47.8 ± 5.7 ^b^	375.4 ± 48.0 ^C^	758.8 ± 9.0 ^A^	685.1 ± 35.7 ^B^	619.4 ± 47.6 ^B^
41	1-propanol	71,238	544.8	121.5	1.11	423.6 ± 23.5 ^a^	451.5 ± 54.2 ^a^	281.9 ± 89.5 ^b^	390.0 ± 23.4 ^a^	737.4 ± 50.1 ^C^	1526.4 ± 119.3 ^B^	1660.4 ± 71.8 ^AB^	1779.3 ± 77.5 ^A^
43	ethanol	64,175	458.9	98.4	1.13	223.0 ± 7.0 ^b^	178.7 ± 10.5 ^c^	174.3 ± 23 ^c^	6116.3 ± 9.1 ^a^	5175.2 ± 243.1 ^A^	856.6 ± 45.6 ^B^	626.8 ± 30.8 ^B^	719.8 ± 19.4 ^B^
47	pentan-1-ol-D	71,410	767.6	192.3	1.51	23.9 ± 2.2 ^a^	23.7 ± 2.6 ^a^	24.9 ± 3.7 ^a^	23.5 ± 4.8 ^a^	275.6 ± 51.3 ^C^	959.1 ± 94.5 ^C^	1040.5 ± 61.5 ^B^	1286.2 ± 12.3 ^A^
Ketones													
18	2-heptanone	110,430	891.6	256.6	1.23	80.1 ± 7.0 ^a^	59.5 ± 2.9 ^b^	87.2 ± 12.1 ^a^	58.4 ± 8.2 ^b^	275.7 ± 35.1 ^C^	508.4 ± 71.9 ^B^	736.9 ± 20.6 ^A^	443.5 ± 8.6 ^B^
31	2-Pentanone-M	107,879	687.7	160.1	1.12	285.4 ± 11.6 ^b^	222.5 ± 8.2 ^b^	215.0 ± 25.8 ^b^	653.4 ± 74.7 ^a^	121.7 ± 20.7 ^A^	63.7 ± 1.4 ^B^	63.8 ± 1.5 ^B^	66.6 ± 2.4 ^B^
32	2-Pentanone-D	107,879	682.7	158.7	1.37	246.7 ± 16.2 ^a^	104.9 ± 14.0 ^c^	137.6 ± 10.1 ^b^	87.9 ± 17.4 ^c^	352.9 ± 15.5 ^C^	607.6 ± 30.5 ^A^	514.0 ± 7.7 ^B^	588.7 ± 7.3 ^A^
40	2-Butanone	78,933	575.7	129.9	1.25	481.6 ± 21.0 ^a^	358.4 ± 35.0 ^b^	319.7 ± 17.8 ^b^	270.5 ± 21.9 ^c^	841.9 ± 55.7 ^C^	1195.0 ± 55.3 ^B^	1258.2 ± 34.7 ^B^	1350.8 ± 7.1 ^A^
42	acetone	67,641	512.8	112.9	1.12	574.7 ± 56.6 ^b^	356.8 ± 43.1 ^c^	430.4 ± 57.2 ^c^	1408.8 ± 89.2 ^a^	1540.5 ± 79.8 ^AB^	1646.2 ± 119.2 ^A^	1291.5 ± 30.0 ^D^	1394.9 ± 58 ^BC^
45	3-hydroxybutan-2-one	513,860	715.1	171.0	1.33	382.9 ± 44.0 ^b^	266.6 ± 39.9 ^b^	63.4 ± 6.3 ^c^	707.6 ± 119.7 ^a^	79.0 ± 12.4 ^C^	137.1 ± 9.0 ^B^	198.8 ± 4.1 ^A^	184.9 ± 12.4 ^A^
54	2,3-butanedione	431,038	576.7	130.1	1.17	43.8 ± 9.3 ^b^	50.7 ± 4.2 ^b^	68.2 ± 19.6 ^b^	449.1 ± 29.1 ^a^	422.7 ± 26.7 ^A^	45.0 ± 10.2 ^B^	44.3 ± 2.2 ^B^	48.4 ± 3.4 ^B^
19	isoamyl acetate	123,922	880.3	250.6	1.31	62.5 ± 25.8 ^a^	39.0 ± 12.1 ^a^	60.0 ± 14.0 ^a^	44.4 ± 12.3 ^a^	68.5 ± 15.1 ^A^	91.8 ± 17.0 ^A^	74.5 ± 3.8 ^A^	82.1 ± 5.9 ^A^
24	butyl propanoate	590,012	905	267.8	1.28	12.9 ± 1.7 ^a^	11.1 ± 1.2 ^ab^	10.6 ± 1.7 ^ab^	8.7 ± 2.3 ^b^	30.7 ± 3.6 ^A^	31.0 ± 8.6 ^A^	31.0 ± 4.5 ^A^	23.8 ± 6.5 ^A^
49	Ethyl formate	109,944	509.8	112.2	1.24	23.7 ± 2.2 ^c^	37.6 ± 4.0 ^b^	49.3 ± 9.3 ^a^	25.3 ± 4.8 ^c^	92.6 ± 9.7 ^B^	71.0 ± 6.7 ^B^	280.2 ± 142.8 ^A^	89.6 ± 1.8 ^B^
53	Ethyl Acetate	141,786	602.7	137.1	1.34	35.0 ± 5.9 ^b^	19.4 ± 2.6 ^b^	27.9 ± 6.6 ^b^	88.0 ± 17.2 ^a^	208.0 ± 46.0 ^A^	97.2 ± 11.8 ^B^	111.6 ± 5.8 ^B^	122.8 ± 17.9 ^B^
Acids													
20	Hexanoic acid	142,621	991.2	341.4	1.30	74.1 ± 12.2 ^b^	66.7 ± 6.2 ^b^	126.0 ± 26.8 ^a^	67.6 ± 6.6 ^b^	61.0 ± 2.1 ^B^	54.2 ± 4.0 ^B^	55.7 ± 2.7 ^B^	72.1 ± 8.2 ^A^
25	2-Methylbutanoic acid	116,530	835.6	226.6	1.21	27.1 ± 4.1 ^bc^	19.8 ± 4.7 ^c^	86.2 ± 40.9 ^a^	62.7 ± 4.8 ^ab^	15.0 ± 0.6 ^A^	17.9 ± 2.3 ^A^	17.2 ± 2.7 ^A^	20.1 ± 5.3 ^A^
55	Butanoic acid	107,926	825.4	221.1	1.16	12.1 ± 0.3 ^a^	15.0 ± 3.0 ^a^	23.7 ± 11.9 ^a^	11.4 ± 1.3 ^a^	13.1 ± 2.3 ^B^	21.8 ± 6.1 ^B^	39.4 ± 4.1 ^A^	41.6 ± 5.2 ^A^
Furans													
23	2-Pentylfuran	3,777,693	995.7	345.2	1.26	22.2 ± 3.6 ^a^	16.4 ± 2.9 ^b^	21.0 ± 0.8 ^ab^	16.5 ± 0.9 ^b^	97.4 ± 17.4 ^C^	205.1 ± 9.6 ^B^	279.0 ± 28.2 ^A^	284.0 ± 8.7 ^A^
Sulfur compounds													
29	Dimethyl disulfide	624,920	745.7	183.5	1.14	36.5 ± 14.0 ^b^	41.9 ± 4.3 ^b^	182.6 ± 91.1 ^a^	148.5 ± 21.4 ^a^	30.7 ± 4.4 ^C^	53.7 ± 2.3 ^AB^	58.9 ± 1.0 ^A^	50.0 ± 4.1 ^B^
Hydrocarbons													
3	Octanal-M	124,130	1006.8	360.8	1.40	262.7 ± 26.0 ^a^	200.1 ± 32.0 ^b^	260.2 ± 27.0 ^a^	184.2 ± 14.0 ^b^	1215.8 ± 198.3 ^C^	2396.4 ± 93.9 ^B^	2648.9 ± 69.7 ^A^	2368.9 ± 60.5 ^B^
4	Octanal-D	124,130	1004.3	357.1	1.83	70.6 ± 5.3 ^a^	75.8 ± 11.3 ^a^	70.5 ± 7.2 ^a^	71.9 ± 5.9 ^a^	354.2 ± 134.2 ^C^	1629.9 ± 151.2 ^B^	2355.7 ± 129.7 ^A^	2220.2 ± 104.4 ^A^

Notes: M, monomer; D, dimer; Rt, retention time in the capillary GC column; RI, retention index calculated using *n*-ketones C4–C9; Dt, rift time in the drift tube. a–d: different letters indicate significant differences of raw groups between laughter methods (*p* < 0.05). A–D: different letters indicate significant differences of steaming groups between slaughter (*p* < 0.05).

**Table 3:** Sensors and sensitive compounds of electronic nose.

Sensors	Sensitive Compounds
LY2/LG	Oxynitride, sulfide, chloride, fluorine
LY2/G	Carbon oxide, amines, ammonia
LY2/AA	Ammonia, ethanol, acetone
LY2/GH	Amines, ammonia
LY2/gCTL	Hydrogen sulfide
LY/gCT	Propane, butane
T30/1	Chloride
P10/1	Hydrocarbon, ammonia, chlorine
P10/2	Methane, ethane
P40/1	Chlorine, fluorine
T70/1	Toluene, xylene, carbon oxide
PA/2	Amines, ammonium hydroxide, ethanol
P30/1	Hydrocarbon, ammonia, ethanol
P40/2	Hydrogen sulfide, chlorine, fluorine
P30/2	Ketone, hydrogen sulfide
T40/2	Chlorine, fluorine
T40/1	Fluorine
TA/2	Ethanol

**Table 4:** Sensory evaluation standard for steaming sea bass.

Evaluation Project	Evaluation Content	Evaluation Standard	
Flavor	Whether there is an inherent flavor of sea bass, and whether there is any peculiar flavor	Samples have a strong chicken flavor and taste, a unique umami taste of soft-boiled chicken and no bloody taste	9
		Samples have a light umami taste, a little bit of peculiar, delicious flavor and slightly bloody taste	5
		The samples have a bloody taste without the unique umami taste and flavor	1
Texture profile	Based on the intuitive steaming sea bass quality and state of the skin during the oral processing	The sample has elastic, tender skin	9
		The sample is tender, but the elasticity is weak	5
		The texture of the sea bass is like chewing wax, and without elasticity	1
Comprehensive scores	Preference	Highly like	9
		Average	5
		Highly dislike	1

## Data Availability

The data presented in this study are contained within the article.

## Sample Availability

Samples of the compounds are available from the authors.
